# When It’s Not Pancreatitis, Don’t Brush It Off: A Case Report of Small Bowel Perforation Caused by a Grill Brush Bristle Masquerading As Pancreatitis

**DOI:** 10.7759/cureus.30422

**Published:** 2022-10-18

**Authors:** Maha Sulieman, Mary Ann Kirkconnell Hall, Gayle Wong, Reem Ahmed

**Affiliations:** 1 Hospital Medicine, Emory University School of Medicine, Atlanta, USA

**Keywords:** foreign bodies ingestion, abdominal foreign body, pancreatitis, magnetic resonance imaging, small bowel perforation

## Abstract

Injuries caused by grill brush bristle ingestion have been documented in the literature, but most existing literature focuses on consumer safety and increasing public awareness of potential injuries. Small bowel perforation is a serious complication and often difficult to diagnose since symptoms are frequently nonspecific and bristle localization can be challenging. We highlight a case where a diagnosis of acute pancreatitis was initially made by computerized tomography (CT) imaging but was later determined to be small bowel perforation with magnetic resonance imaging (MRI).^1 ^Due to its high resolution and excellent anatomic depiction of different pathologies, including inflammation and tumors, MRI is often used as an imaging modality when the cause of pancreatitis is not clear through initial history, physical exam, or imaging modalities like ultrasound and CT scan. MRI provides an opportunity to detect pathologies that cannot be depicted by CT because of its high contrast resolution (though conversely, CT has a higher spatial resolution, so there are some cases in which it can detect things that MRI cannot). This case highlights the importance of considering MRI to diagnose and evaluate complications in suspected cases of wire bristle ingestion prior to endoscopic or surgical extraction.

## Introduction

Wire brushes are a common tool used for cleaning grills, but brush bristles can dislodge, adhere to food, and be accidentally ingested, causing serious injuries like intestinal perforation in our patients [1,2]. A number of recent cases have been reported in the literature, particularly over the last decade, and grill brush bristles were added to the Consumer Product Safety Commission’s https://www.saferproducts.gov database in 2011 [3,4]. The diagnosis of grill brush bristle ingestion is challenging, as symptoms are often nonspecific, bristle localization can be difficult, and when ingestion does occur, injuries are most frequently found in the oral cavity or oropharynx rather than further down the alimentary tract [3]. Patients are usually unaware of the ingestion, which sometimes delays presentation [5]. Nausea, vomiting, and abdominal pain are common, but globus sensation and odynophagia along with a history of grilled food consumption may also suggest the diagnosis [6].

We describe the utility of magnetic resonance imaging (MRI) as an investigatory tool for determining the true source of perforation in a case initially suspected to be pancreatitis, but which lacked a number of clinical features typically associated with the diagnosis. MRI is increasingly used to diagnose a variety of pancreatic diseases in cases of clinical uncertainty [7]; in this case, MRI was deemed to be more specific than computerized tomography (CT) scan in determining the correct diagnosis for the patient. 

## Case presentation

The patient was a 72-year-old male with a medical history of hypertension, paroxysmal atrial fibrillation, gastroesophageal reflux disease, and hyperlipidemia. He maintained a healthy lifestyle, exercised regularly, and did not consume tobacco or alcohol. He presented with fever and epigastric pain for three days. He described his epigastric pain as constant and dull, to the left side of the midline. He initially attributed his symptoms to possible food poisoning, as he recalled eating restaurant food prior to feeling sick; however, his pain became more persistent, and he presented to the hospital. Table 1 depicts a timeline of his case.

**Table 1:** Case Timeline

Hospital Day	Significant Events
-3	Onset of epigastric pain.
0	Patient presents with fever and worsening epigastric pain. CT demonstrated pancreatitis; the patient was started on conservative management for acute pancreatitis.
1	Patient continued to have abdominal pain, which improved temporarily with IV narcotics. Hepatobiliary scan and immunoglobulin subtype 4 were done as etiology of pancreatitis was not clear; results were negative. Esophagogastroduodenoscopy was conducted and was unrevealing.
2	Pain persisted, and the patient expressed that he felt unwell. Magnetic resonance cholangiopancreatography was performed to further evaluate the pancreas/etiology of pancreatitis. MRI showed a foreign body perforating the duodenal wall in the third portion of the duodenum. The patient was started on IV antibiotics. Gastroenterology decided on push enteroscopy to attempt extraction; surgical oncology evaluated and concurred with the GI plan.
3	Esophagogastroduodenoscopy/push enteroscopy was done and the foreign body was extracted. The patient’s pain improved.
4	Patient was discharged home with oral antibiotics.

Clinical Findings

**Figure 1:**
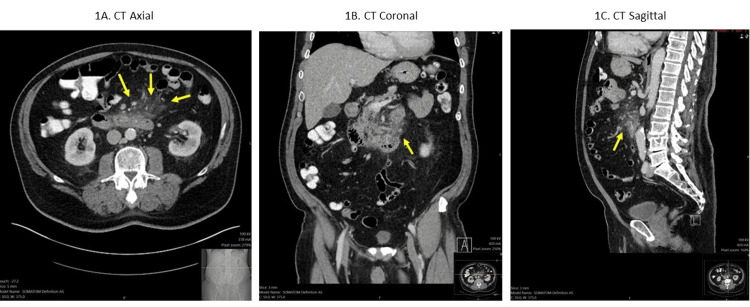
Axial (1A), coronal (1B), and sagittal (1C) CT scans of the abdomen and pelvis with IV contrast showing stranding and inflammation around the duodenum and pancreas, read as pancreatitis/duodenitis. Note the inflammation obscuring the visualization of the grill bristle brush in the third portion of the duodenum in Figures 1A and 1B, making delineation of the foreign body by CT challenging.

Physical exam on presentation was significant for epigastric and left periumbilical tenderness but otherwise normal. He was febrile with a temperature of 38.4° F and blood pressure of 126/80 mmHg, with normal heart rate and oxygen saturation. Laboratory data revealed elevated white blood cells (18.0/μL), elevated inflammatory markers (ESR 39 mm/hr, CRP 134.80 mg/L), normal lipase of 27 unit/L, and a triglyceride level of 94 mg/dl. A CT scan of his abdomen and pelvis with intravenous (IV) contrast showed peripancreatic fat stranding around the head and the uncinate process of the pancreas, suggesting acute pancreatitis (Figure 1). It also showed inflammatory stranding adjacent to the third part of the duodenum, which was thought to be related to the pancreatic process. He was started on conservative management with bowel rest, IV fluid administration, and pain medications.

Diagnostic Assessment

Despite conservative management, the patient continued to have abdominal pain. His gallbladder appeared normal on CT and subsequently on a hepatobiliary scan (using radioisotope inspection with intravenous administration of 9.0 mCi of technetium and 99m trimethyl bromide). There was no evidence of cystic duct obstruction. Abdominal ultrasound showed poor visualization of the pancreas. Otherwise, the exam findings were unremarkable. Immunoglobulin subtype 4(Ig-G4) was normal, signifying low suspicion of autoimmune pancreatitis.

The patient’s normal lipase level, lack of risk factors for pancreatitis including alcohol consumption, absence of gallstones, and persistence of symptoms prompted the evaluation of other etiology of his pain. Amylase was not ordered; in a number of studies, lipase has generally been found to be both more sensitive and more specific than amylase for the diagnosis of acute pancreatitis [8]. Esophagogastroduodenoscopy (EGD) was pursued next and showed patchy erythematous mucosa without bleeding in the gastric antrum. The proximal part of the third portion of the duodenum was examined during EGD and was normal. No lesions were observed anywhere in the duodenum.

**Figure 2:**
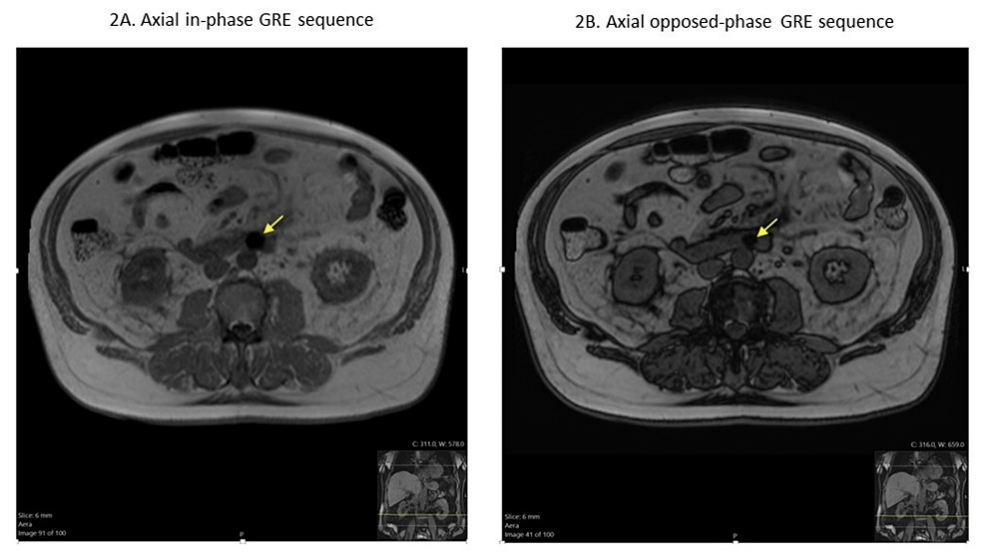
MRI of the abdomen and pelvis. Both sequences show signal loss (aka susceptibility artifact). More signal loss on in-phase (2A) compared to opposed phase (2B, known as blooming artifact) is indicative of paramagnetic material (weakly magnetic metals).

**Figure 3:**
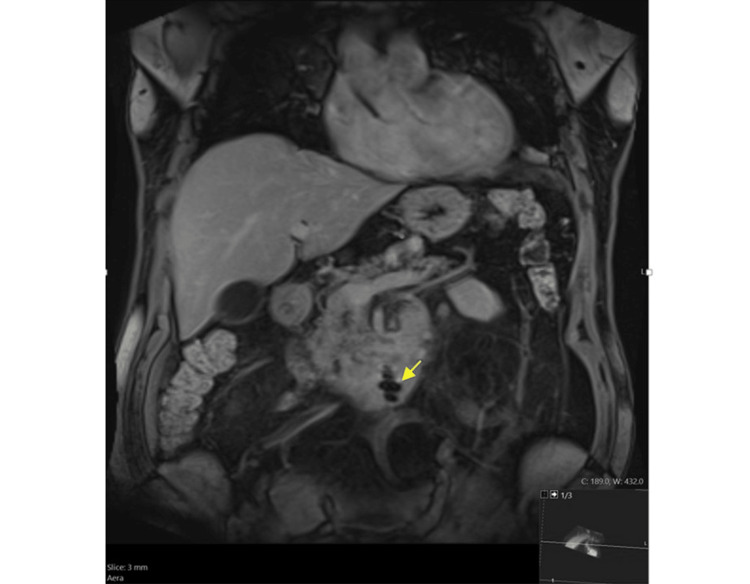
Coronal VIBE post contrast: Susceptibility artifact in the distal horizontal duodenum indicative of the foreign body.

An MRI of the abdomen with and without contrast (Figure 2) was then performed and showed a perforating duodenal foreign body (possibly a fishbone; Figure 3) with transmural extension into adjacent mesentery, causing marked focal duodenitis, peri-duodenal phlegmon, and adjacent reactive lymphadenopathy. There was no evidence of pancreatitis on the MRI.

Therapeutic Intervention

**Figure 4:**
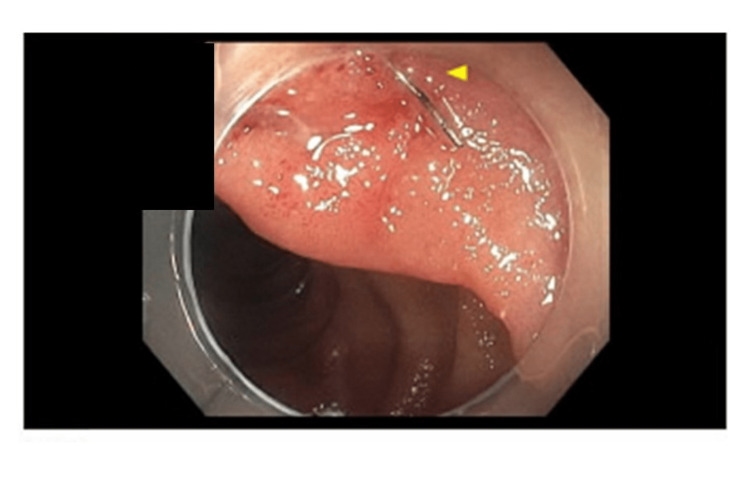
Small bowel push enteroscopy: the bristle in the third portion of the duodenum.

**Figure 5:**
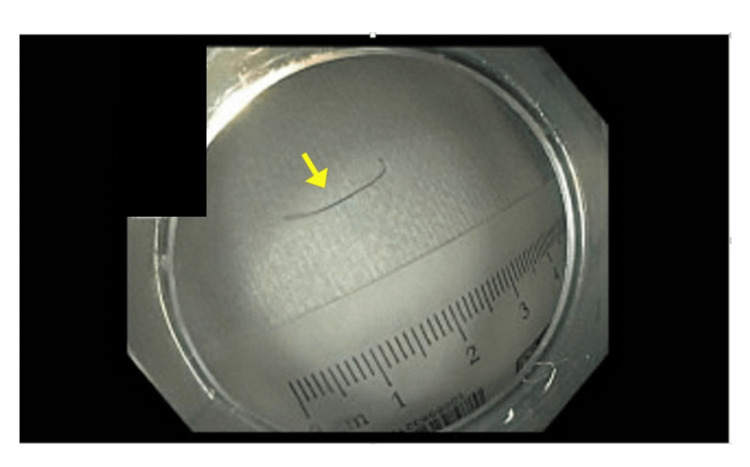
Grill brush bristle after removal from the patient's duodenum.

The new findings on MRI changed the working diagnosis completely and shifted the therapeutic focus from conservative to interventional management. Extraction of the foreign body identified in images became a priority. The patient, who had started on an oral diet at the time, was placed back on nil per os, or nothing by mouth (NPO) and was started on IV antibiotics with piperacillin-tazobactam. Surgical consultation recommended a trial of endoscopic removal, and if unsuccessful, consideration of open surgical extraction. A small bowel enteroscopy was performed and revealed a few localized erosions with no bleeding in the duodenal bulb and the second portion of the duodenum (Figure 4). A foreign body in the third portion of the duodenum was successfully removed with a snare. Upon close inspection, the foreign body was identified as a steel bristle from a grill brush (Figure 5). The patient recalled and reported ingestion of grilled food prior to symptom development.

Follow-Up and Outcome

The patient’s symptoms improved greatly after the extraction, and with the addition of antibiotics, his pain became intermittent and he was able to tolerate a regular diet. He required additional non-surgical treatment with extended antibiotic therapy with amoxicillin/clavulanic acid and close follow-up after discharge and eventually recovered completely.

## Discussion

In the adult population, perforation of the small bowel caused by wire-bristle ingestion appears to be a relatively common injury, likely due to the bristle’s small diameter and frequent angulation within the small bowel, though it is observed less frequently than oral or oropharyngeal injury [3]. Delays in diagnosis and removal of foreign bodies in the gastrointestinal tract can cause significant morbidity and mortality; consequences include perforation, bleeding, abscess, and fistula formation [5].

Depending on the site of penetration, the clinical presentation may mimic more common medical conditions, e.g., peptic ulcer disease or gastritis if the bristle lodges in the stomach, or pancreatitis if the bristle is in the pancreas or duodenum [9]. Micro-perforations of the small bowel are usually subtle and subacute in nature and present with less severe symptoms than perforations in other parts of the gastrointestinal tract [10-12]. Because the management of common mimic conditions is mostly conservative, high clinical suspicion is necessary when clinical data is not consistent with the presumed diagnosis, as in our case. Radiological and or endoscopic diagnosis is essential for the evaluation of suspected foreign body ingestion in these cases [1,5].

CT scans are the preferred method of pre-procedural diagnosis and are recommended for initial localization and evaluation of complications, including bowel perforation [13]. The use of IV contrast enhances bowel wall layers and can obscure foreign objects [1,14]. Non-contrast CT may be more sensitive in detecting the intrinsic contrast of the foreign body against the nonenhanced bowel wall; however, this usually requires a pre-imaging high clinical suspicion of foreign body ingestion [14].

Due to the small amount of free extraluminal air in small bowel perforation relative to the perforation of other parts of the gastrointestinal tract, a CT scan may fail to detect small bowel perforation [15]. Free air is not detected in 50% of small bowel perforation cases on CT scans [15]. Intestinal wall leakage, if oral contrast is used, can aid in diagnosis. In cases like our patient, CT may fail to detect the bristle and/or the micro-perforation [10].

MRI has excellent soft tissue resolution [16]. Findings such as mural edema and severe inflammation can be evaluated on T2-weighted and diffusion-weighted images, respectively [17]. Magnetic resonance cholangiopancreatography is particularly important in the evaluation of the pancreas and biliary tract when initial images suggest a mimicker, like pancreatitis in our case. Despite the higher cost and time needed for MRI, it is considered a less invasive localization method than endoscopic evaluation [17].

Multiple endoscopic versus surgical procedures may be required for localization and safe removal [6]. Brooks et al. (2019) discussed how the use of intraoperative ultrasound in the precise localization and successful extraction of ingested wire bristles can be superior to standard preoperative radiological testing, including CT and MRI [18]. While useful, intraoperative ultrasound utilization is limited in cases with contained small bowel perforation and when no surgical repair is warranted [19]. Surgical exploration is more invasive than endoscopic removal; MRI can thus be considered a more appropriate method for detection and localization [19].

## Conclusions

In conclusion, we highlight a case where MRI was utilized to diagnose a grill bristle perforation and exclude other pancreatic pathology. This case also highlights the importance of recognizing alternative modalities such as an MRI to diagnose and evaluate complications in suspected cases prior to endoscopic or surgical extraction.
